# Porous CuO Microspheres as Long-Lifespan Cathode Materials for Aqueous Zinc-Ion Batteries

**DOI:** 10.3390/nano14131145

**Published:** 2024-07-03

**Authors:** Yuqing Ai, Qiang Pang, Xinyu Liu, Fangyun Xin, Hong Wang, Mingming Xing, Yao Fu, Ying Tian

**Affiliations:** School of Science, Dalian Maritime University, Dalian 116026, China; aiyuqing@dlmu.edu.cn (Y.A.); l2829985882@dlmu.edu.cn (X.L.); xin_2020@dlmu.edu.cn (F.X.); hongwang@dlmu.edu.cn (H.W.); mingmingxing@dlmu.edu.cn (M.X.); fuyao@dlmu.edu.cn (Y.F.); tianying@dlmu.edu.cn (Y.T.)

**Keywords:** cathode material, copper oxide, microsphere, zinc-ion battery

## Abstract

Cathode materials with conversion mechanisms for aqueous zinc-ion batteries (AZIBs) have shown a great potential as next-generation energy storage materials due to their high discharge capacity and high energy density. However, improving their cycling stability has been the biggest challenge plaguing researchers. In this study, CuO microspheres were prepared using a simple hydrothermal reaction, and the morphology and crystallinity of the samples were modulated by controlling the hydrothermal reaction time. The as-synthesized materials were used as cathode materials for AZIBs. The electrochemical experiments showed that the CuO-4h sample, undergoing a hydrothermal reaction for 4 h, had the longest lifecycle and the best rate of capability. A discharge capacity of 131.7 mAh g^−1^ was still available after 700 cycles at a current density of 500 mA g^−1^. At a high current density of 1.5 A g^−1^, the maintained capacity of the cell is 85.4 mA h g^−1^. The structural evolutions and valence changes in the CuO-4h cathode material were carefully explored by using ex situ XRD and ex situ XPS. CuO was reduced to Cu_2_O and Cu after the initial discharge, and Cu was oxidized to Cu_2_O instead of CuO during subsequent charging processes. We believe that these findings could introduce a novel approach to exploring high-performance cathode materials for AZIBs.

## 1. Introduction

In the last few years, the field of rechargeable aqueous zinc-ion batteries (AZIBs) has attracted the attention of many researchers and has achieved significant technological advances [1,2,3]. These batteries utilize an aqueous electrolyte, which not only ensures a high level of system safety, but also maintains a low-cost advantage. Zinc metal anodes are favored for their large storage capacity, offering a theoretical capacity of 820 mA h g^−1^, highlighting their potential for energy storage [4]. As for cathode materials, most research has focused on intercalation/de-intercalation-type materials. The redox reactions in these materials involve processes of Zn^2+^ ion and/or proton intercalation and de-intercalation in weakly acidic electrolytes [5,6]. In particular, manganese oxides [7,8] and vanadium oxides [9,10] are studied quite intensively as electrode materials for AZIBs. Other types of compounds, such as Prussian blue analogs [11] and polyanions [12], are considered as possible candidate cathode materials to achieve improved battery performance. The cathode materials with conversion mechanisms exhibit a distinctive electrochemical reaction mechanism, characterized by their capacity to break and rearrange chemical bonds during electrochemical reactions, facilitating charge transfer [13]. During this process, the material typically undergoes a phase change phenomenon, where the cathode material experiences reversible structural and compositional changes throughout the discharge and charge processes. Usually, conversion-type cathode materials have a higher reversible capacity than conventional intercalation/de-intercalation-type cathodes.

Recently, some conversion-type cathode materials have been shown to be suitable for AZIBs, mainly including chalcogens (S [14], Se [15], and Te [16]), halogens (I_2_ [17] and Br_2_ [18]), and transition metal oxides (MnO_2_ [19] and CuO [20,21,22,23]). These materials undergo multi-electron transfer reactions during charge and discharge, thus possessing a high theoretical capacity. For example, the theoretical capacity of S is 1675 mA h g^−1^. However, the generation of inactive by-products and the dissolution of sulfides can cause the rapid capacity decay of Zn-S batteries [24]. In addition, the poor electronic conductivity of S brings a significant voltage hysteresis to the Zn-S battery, resulting in a low operating voltage and a poor energy efficiency [25]. To enhance the cycling stability of the S cathode for AZIBs, it is mainly combined with carbon-based materials to improve electronic conductivity and to prevent the loss of active materials [26]. As a representative of the halogen-based cathodes, I_2_ is famous for its multi-form of valence states such as I_2_, I^−^, I_3_^−^, and I_5_^−^ [27], which means that Zn-I_2_ batteries exhibit a high theoretical capacity and multiple discharge voltage plateaus. Unfortunately, the soluble I_3_^−^ and I_5_^−^ species can dissolve into the electrolyte and react with the zinc metal anode, the so-called shuttle effect, leading to the performance degradation of Zn-I_2_ batteries [28]. The MnO_2_ cathode is favored for its high capacity and low production cost. It is demonstrated that a conversion reaction between MnO_2_ and MnOOH with a practical reversible capacity of more than 200 mA h g^−1^ and an average discharge voltage of about 1.2 V occurs in a Zn-MnO_2_ battery [29]. However, the dissolution of Mn severely shortens the cycle life of the battery, although adding manganese sulfate to the electrolyte in advance can alleviate the capacity reduction to some extent [30]. Compared with other conversion-type materials, CuO has the advantages of abundant resources, environmental friendliness, and easy preparation. As a battery electrode material, CuO exhibits a good electronic conductivity and a satisfactory theoretical capacity of 674 mA h g^−1^ [31]. The conversion mechanism of the Zn-CuO battery has been reported by Meng et al. in 2020 [32]. It is also demonstrated that the CuO cathode undergoes continuous active material loss and structural degradation with a capacity decrease during cycling. Although the cycle life of the Zn-CuO batteries can be improved by adjusting the electrolyte composition or optimizing the electrode structure [33,34], it is still a big challenge for researchers to design and synthesize high-performance CuO cathode materials for AZIBs.

In this work, porous CuO microspheres assembled from nanorods were successfully prepared and were first utilized as cathode material for AZIBs. The effects of different hydrothermal reaction times during the preparation process on the structural, morphological, and electrochemical properties of the samples were investigated. The porous microsphere morphology of CuO enables the material to possess a large specific surface area and enhanced charge transfer kinetics. The results showed that the CuO-4h sample, synthesized with a hydrothermal reaction time of 4 h, exhibited the best cycling stability and rate capability. The battery still had a high discharge capacity of 131.7 mA h g^−1^ with a capacity retention of 87.6% after 700 cycles at a current density of 0.5 A g^−1^. At a high current density of 1.5 A g^−1^, the discharge capacity was still 85.4 mA h g^−1^. Compared to other CuO cathodes reported recently [21,22,23,32], the as-prepared CuO microspheres in this work show a hierarchical porous structure and a microsphere morphology. This allows the material to have a highly compacted density, while ensuring electrolyte infiltration. In addition, the CuO microspheres show a better cycling stability and an improved rate performance.

## 2. Materials and Methods

### 2.1. Preparation Method of CuO Microspheres

In a typical synthesis, 0.998 g of CuSO_4_·5H_2_O (Sinopharm Chemical Reagent Co., Ltd., Beijing, China) was dissolved in 50 mL of deionized water and was stirred for 10 min to obtain a homogeneous solution. Then, 0.6 mol of Na_2_CO_3_ (Sinopharm Chemical Reagent Co., Ltd., Beijing, China) was weighed and added to 20 mL of deionized water and was stirred until completely dissolved. This solution was slowly dropped into the former solution and stirred at room temperature for 1 h. The obtained suspension was transferred into a polytetrafluoroethylene autoclave liner with a volume of 100 mL, and was then sealed with a stainless steel autoclave jacket and put in an oven to carry out a hydrothermal reaction at 200 °C. The control of the holding time was 2 h, 4 h, and 6 h, respectively. The reactor cooled naturally to room temperature, and the reaction products were collected and washed with deionized water and ethanol via centrifugation several times; the final products were dried in an oven at 60 °C for 24 h. The samples with heating times of 2 h, 4 h, and 6 h were named CuO-2h, CuO-4h, and CuO-6h, respectively.

### 2.2. Material Characterizations

X-ray diffraction (XRD) patterns were acquired using an XRD-6000X diffractometer (Tokyo, Japan) with Cu Kα radiation for the CuO-2h, CuO-4h, and CuO-6h powders, employing a D/MAX-Ultima diffractometer with Co Kα radiation for the electrodes. For scanning electron microscopy (SEM), a SUPRA 55 SAPPHIRE field-emission microscope (Oberkochen, Germany) was used to observe the morphology and particle size of the samples. Transmission electron microscopy (TEM) photographs of the sample were acquired using a JEOL JEM-2100 transmission electron microscope (Tokyo, Japan). The N_2_ adsorption/desorption isotherms were measured on an ASAP 2460 Micromeritics (Norcross, GA, USA). The element valences of the samples were detected using a K-Alpha X-ray photoelectron spectrometer (Waltham, MA, USA). For the XRD and XPS tests of the electrodes, the coin-type batteries were disassembled with a plier, washed with deionized water carefully, and were dried at 70 °C before use.

### 2.3. Electrochemical Experiments

First, 60 wt.% CuO powder, 30 wt.% vapor growth carbon fiber (VGCF, resistivity is about 0.01 Ω·m, provided by the manufacturer (SAIBO Electrochemical Material Co., Ltd., Shenzhen, China)), and 10 wt.% polyvinylidene difluoride (PVDF) were mixed well in the mortar. The mixed powder was uniformly dispersed in a N-methyl pyrrolidone (NMP) solvent to form a slurry. This slurry was evenly coated on a Ti metal foil. After drying at 60 °C overnight, the Ti metal foil was cut into circles. The radius of the circle is 4 mm, and the mass of active material (CuO) on the electrode was about 0.5 mg. The electrolyte used in this study was an aqueous solution of zinc sulfate, and the concentration was 3 mol L^−1^. A zinc metal sheet measuring 1 by 1 cm was used as the anode. The separator was a circular Whatman GF/C glass fiber filter paper cut into a radius of 9 mm. The coin-type (CR2032) cells were packaged using an MSK-110 hydraulic battery sealing machine (Hefei, China). The galvanostatic charge/discharge (GCD) tests of the batteries were conducted on a LAND CT2001 system (Wuhan, China). Cyclic voltammetry (CV) of the cells was performed using a CHI 660E electrochemical workstation at a scan rate of 0.1 mV s^−1^. Electrochemical impedance spectroscopy (EIS) tests were performed on a CHI660E electrochemical workstation (Shanghai, China) in the frequency range from 1 MHz to 0.1 Hz. The voltage perturbation amplitude of the EIS tests was 10 mV. The EIS spectra were obtained after the battery was discharged to 0.4 V and was stood for 4 h.

## 3. Results and Discussion

**Figure 1:**
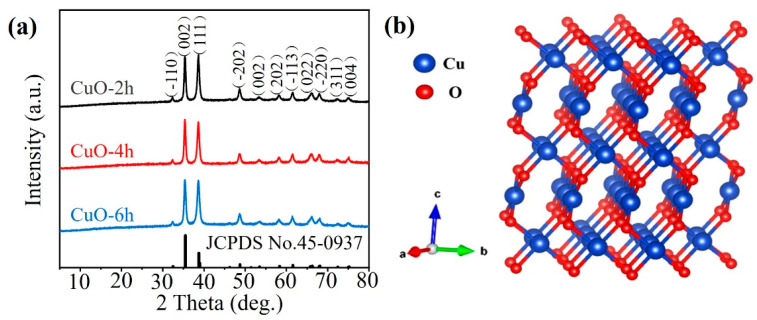
(**a**) XRD curves of the CuO-2h, CuO-4h, and CuO-6h samples. (**b**) A schematic of the crystal structure of CuO.

Figure 1a shows the XRD patterns of the prepared CuO-xh (x = 2, 4, 6) samples. The characteristic XRD peaks of the samples are in accordance with the data of the standard card (JCPDS No.: 45-0937) and no other impurity peaks are observed, which indicates that the synthesized CuO samples are well-crystallized and are of high purity. Figure 1b displays a model of the crystal structure of CuO simulated using the VESTA software (Ver. 3.5.8). CuO is composed of stacks of parallelograms connected by O-O bonds. A single CuO unit is a parallelogram unit consisting of a Cu atom at the center and four O atoms connected to form an O-O bond, where the Cu atom adopts a tetra-coordinated configuration, while each crystal cell consists of four CuO units. The spacing of the crystal faces of CuO (111) is 3.48 Å, which is much larger than the ionic radius of Zn^2+^ (0.74 Å) and can be favorable in the electrochemical reaction for Zn^2+^ intercalation and de-intercalation, and also helps reduce the volume expansion caused by the reaction process.

**Figure 2:**
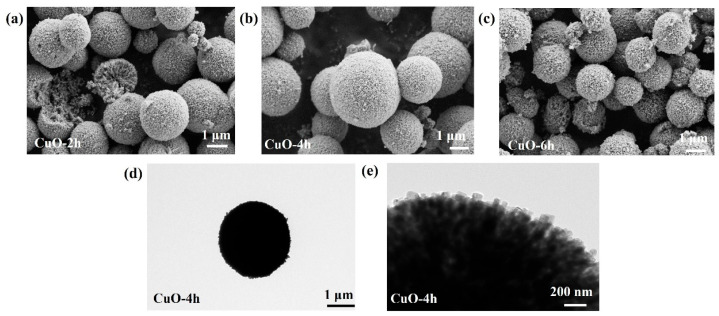
SEM photographs of (**a**) CuO-2h, (**b**) CuO-4h, and (**c**) CuO-6h. (**d**,**e**) TEM photographs of the CuO-4h sample.

Figure 2a–c show the SEM photographs of the CuO-2h, CuO-4h, and CuO-6h samples, respectively, from which it can be seen that the CuO microspheres are constructed by stacking a large number of nanorods together. The diameter of the CuO-2h sample (Figure 2a) is not uniform (1 μm–3 μm) and some microspheres are not spherical, which may be due to the incomplete reaction because of the short reaction time. In contrast, the CuO-4h sample is a perfectly spherical shape (Figure 2b) and the size of the microspheres is more uniform. The CuO-6h sample has some stray particles on the surface of the microspheres (Figure 2c), which may be due to the structural disruption caused by the excessively long reaction time. Figure 2d shows the low-magnification TEM photograph of the CuO-4h sample, from which the spherical morphology with a diameter of approximately 3 μm can be seen. Figure 2e shows a high-magnification TEM photograph of the CuO-4h sample, which clearly shows that the CuO microsphere is assembled from a large number of nanorods with a width of about 50 nm. The lighter-colored spots in the photograph indicate the existence of abundant mesopores inside the CuO microspheres. These morphological features are favorable for the wettability of the electrode and the transport of Zn^2+^.

**Figure 3:**
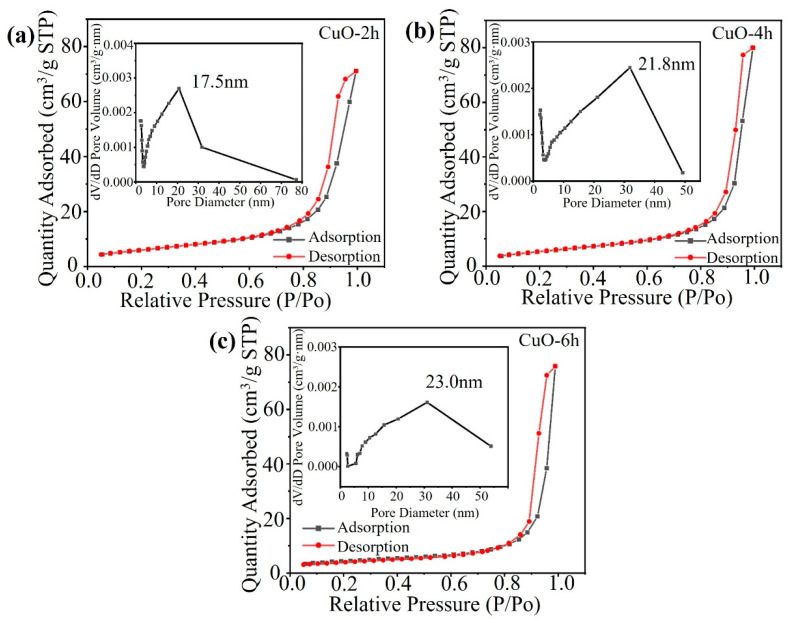
N_2_ adsorption/desorption isotherm plots of (**a**) CuO-2h, (**b**) CuO-4h, and (**c**) CuO-6h. Insets are the pore-size distributions of the samples.

As shown in Figure 3, to further confirm the pore structure inside the sample particles, the specific surface area and pore size of the CuO-2h, CuO-4h, and CuO-6h samples were characterized using N_2_ adsorption–desorption tests. It can be seen that the N_2_ adsorption/desorption isotherms of the three samples belong to the type IV physical adsorption isotherms according to the IUPAC classification. They display a more obvious adsorption plateau, which is typical of mesoporous structures. The specific surface areas of the CuO-2h, CuO-4h, and CuO-6h samples are 15.4 m^2^ g^−1^, 19.6 m^2^ g^−1^, and 21.7 m^2^ g^−1^, respectively, and the average pore sizes of the three samples are 17.5 nm, 21.8 nm, and 23.0 nm, respectively, as calculated using the BJH method (insets of Figure 3a–c), which shows that the pores inside the samples are mainly of a mesoporous structure, and the average pore size and the specific surface area increase with the hydrothermal reaction time.

**Figure 4:**
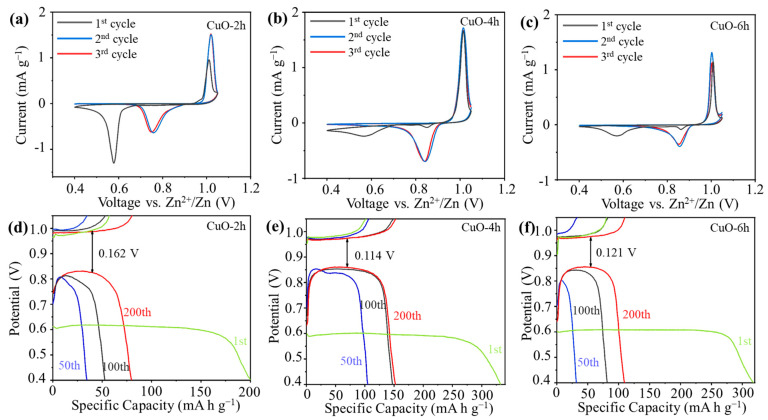
The first three CV curves of (**a**) CuO-2h, (**b**) CuO-4h, and (**c**) CuO-6h at a scan rate of 0.1 mV s^−1^. Charge/discharge profiles of (**d**) CuO-2h, (**e**) CuO-4h, and (**f**) CuO-6h at 500 mA g^−1^.

CV curves of the CuO-2h//Zn, CuO-4h//Zn, and CuO-6h//Zn cells are shown in Figure 4a–c, respectively. The CuO-2h electrode shows a reduction peak at 0.58 V and an oxidation peak at 1.0 V during the first scanning process. The peak at 0.58 V fades away at the second cycle and a new set of peaks emerges at 1.02 V/0.76 V. For the CuO-4h electrode, two reduction peaks at 0.57 V and 0.85 V appear in the first cathodic process and an oxidation peak at 1.0 V appears during the subsequent anodic scan. During the second and third cycles, the reduction peak at 0.57 V disappears, the peak at 0.85 V is enhanced, and the intensity of the oxidation peak at 1.0 V is almost unchanged. The shape and variation of the CV curves of the CuO-6h electrode are similar to those of the CuO-4h electrode, but the intensity of the current density peak is relatively small. The changes in the CV curves of the materials indicate that a structure transition of the electrode materials may have occurred at the end of the first discharge. The overlap of the CV curves after the initial cycle suggests that the CuO-2h, CuO-4h, and CuO-6h electrodes exhibit good reversibility after the initial cycle. The charge/discharge profiles of the electrodes at the 1st, 50th, 100th, and 200th cycles are illustrated in Figure 4d–f. The voltage plateau of the battery for the first discharge is about 0.6 V, which is in agreement with the CV analyses. After about 100 cycles, the discharge voltage plateau of the battery gradually stabilizes at about 0.85 V. The discharge capacities of the CuO-2h//Zn battery for the 1st, 50th, 100th, and 200th cycles are 200.1, 34.6, 52.7, and 79.8 mA h g^−1^, respectively. The capacity is relatively low and decreases quickly. The polarization voltage at the 200th cycle is 0.162 V. The discharge capacities of the CuO-6h//Zn battery at the same cycles are 317.5, 31.5, 81.6, and 109.6 mA h g^−1^, respectively, and the initial capacity is higher; the polarization voltage is 0.121 V. The CuO-4h//Zn battery’s discharge capacities at the 1st, 50th, 100th, and 200th cycles are 332.2, 106.4, 149, and 153.1 mA h g^−1^, respectively, and the cell has the highest capacity and the lowest polarization voltage of 0.114 V compared with the other two batteries.

**Figure 5:**
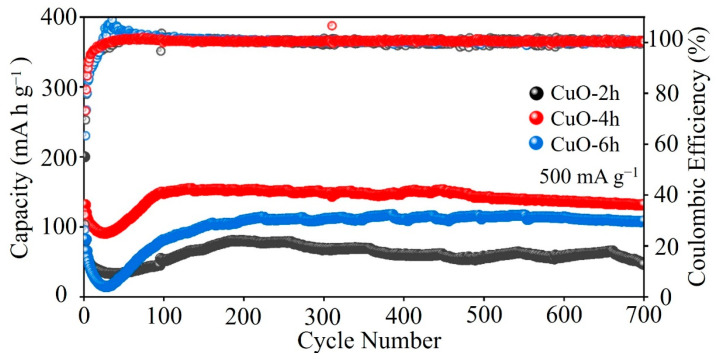
Cycling performance of the CuO-2h, CuO-4h, and CuO-6h electrodes at 500 mA g^−1^.

In order to compare the cycle life of the three cathode materials for AZIBs, we conducted GCD tests on the batteries at a current density of 500 mA g^−1^. As illustrated in Figure 5, the CuO-4h electrode exhibits an initial discharge capacity of 332.2 mA h g^−1^. After about 100 cycles of electrochemical activation, the capacity stabilizes gradually and reaches the highest discharge capacity of 150.3 mA h g^−1^. A high capacity of 131.7 mA h g^−1^ is maintained after 700 cycles with a Coulombic efficiency of approximately 100%. The initial capacities of CuO-2h and CuO-6h are 200.1 mA h g^−1^ and 317.5 mA h g^−1^, respectively. The discharge capacities after 700 cycles are 46.2 mA h g^−1^ and 108.3 mA h g^−1^, respectively. Compared with the cycling performance of the CuO-2h and CuO-6h electrodes, that of the CuO-4h electrodes is much enhanced. This improvement may be attributed to the fact that CuO-4h has a more perfect porous spherical structure. This structure can reduce the volume expansion of the active material and help to maintain the stability of the electrode structure. In addition, the conductive additive used in this work is VGCF. As shown in Figure S1, VGCF has no capacity for AZIBs. Benefiting from the high conductivity and 2D fiber morphology, the battery using VGCF as the conductive additive shows a better cycling stability than the battery using Super P as the conductive additive.

**Figure 6:**
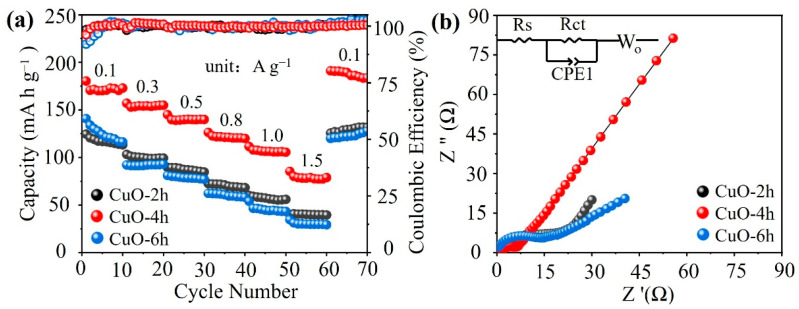
(**a**) Rate performance and (**b**) Nyquist plots of the CuO-2h, CuO-4h, and CuO-6h electrodes.

Figure 6a demonstrates the rate performance of the three electrode materials. It can be seen that the specific capacity of the CuO-4h sample is higher than that of the other two electrodes at all current densities. At current densities of 0.1, 0.3, 0.5, 0.8, 1, and 1.5 A g^−1^, CuO-4h exhibits specific capacities of 180.3, 157, 144.9, 126.3, 111.5, and 85.4 mA h g^−1^, respectively. When the current density returns to 0.1 A g^−1^, the capacity can be recovered up to 191 mA h g^−1^. At the same current densities, the specific capacities of CuO-2h are 116.8, 99.1, 87.3, 71, 57.3, and 39.9 mA h g^−1^ and the capacities of CuO-6h are 124, 91.5, 78.6, 59.5, 44, and 29.7 mA h g^−1^, respectively. To further demonstrate the kinetic properties of the three electrode materials, an EIS test was carried out after the first discharge. As shown in Figure 6b, the semicircles observed in the high-frequency region are due to the charge transfer resistance (R_ct_), while the sloping lines seen in the low-frequency region are due to the diffusion of Zn^2+^ in the electrodes (W_o_), and the intercepts on the real axes represent the ohmic resistance of the cell (R_s_) [35]. By fitting the EIS data using the equivalent circuit in the inset, the R_ct_ value of the CuO-4h electrode is 4.5 Ω, significantly lower than that of the CuO-2h (21.9 Ω) and CuO-6h (10.3 Ω) electrodes. Meanwhile, the slope of the lines in the low-frequency region is correlated with the ion diffusion coefficient of active material [36]. The slope of the CuO-4h electrode is significantly larger than that of the CuO-2h and CuO-6h electrodes. These results indicate that the ion transfer/diffusion kinetics in the CuO-4h electrode are faster. Therefore, the CuO-4h electrode exhibits a better cycling stability and a superior rate capability compared to the other two materials. EIS tests of the batteries after 100 cycles were also carried out (Figure S2). The R_ct_ values of the CuO-2h, CuO-4h, and CuO-6h electrodes are reduced to 20.9 Ω, 2.6 Ω, and 5.8 Ω, respectively. This indicates that the charge transfer kinetics are more favorable after the electrochemical activation process.

**Figure 7:**
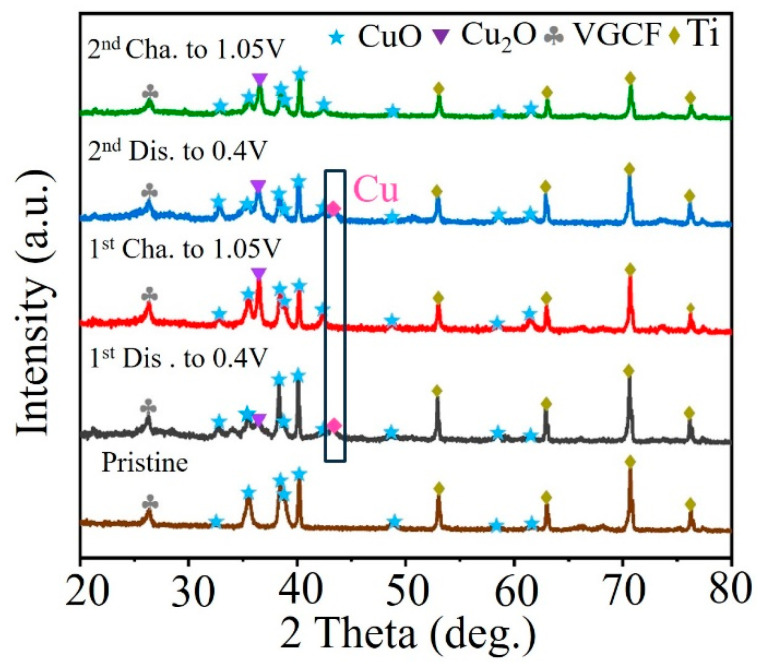
Ex situ XRD patterns of the CuO-4h electrode during the first two cycles.

To identify the structural evolution of CuO during the charge/discharge process, ex situ XRD was performed on the CuO electrode (Figure 7). The XRD pattern of the pristine electrode is identical to that of the synthesized CuO-4h powder, except for the diffraction peaks of the Ti metal current collector and the conductive additive (VGCF). At the first discharge to 0.4 V, the diffraction peaks of CuO are noticeably weakened, and two new diffraction peaks appear at 36.3° and 43° at the same time. The diffraction peak at 36.3° corresponds to the (111) crystalline facet peak of Cu_2_O (purple inverted triangular markers), and the diffraction peak at 43° corresponds to the (111) crystalline facet peak of Cu (pink inverted triangular markers). This indicates that with the embedding of Zn^2+^, the CuO electrode undergoes a conversion reaction to produce Cu_2_O and Cu during the discharge process. When charging to 1.05 V, the diffraction peak of Cu disappears completely, and the intensity of the diffraction peaks of CuO remains almost unchanged, but the peaks of Cu_2_O are significantly enhanced. This suggests that the conversion reaction that occurs during the first charging and discharging process is not completely reversible. Some CuO is reduced to Cu_2_O and Cu during the first discharging process, and Cu is oxidized to Cu_2_O instead of CuO during the subsequent charging process. This result explains the lower Coulombic efficiency of CuO at the initial cycle. The XRD curves during the second cycle at discharging to 0.4 V and charging to 1.05 V show a similar change rule to that of the first cycle, which suggests that the active material involved in the electrochemical reaction during the subsequent electrochemical process is mainly Cu_2_O.

**Figure 8:**
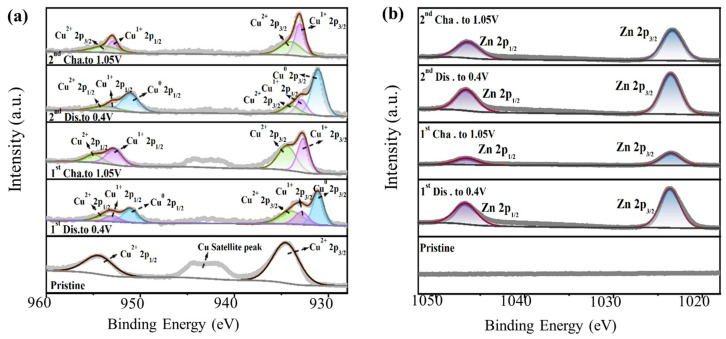
Ex situ XPS spectra of (**a**) Cu and (**b**) Zn during the first two cycles.

Additionally, we also analyzed the valence changes in Cu and Zn by studying the XPS spectra of the electrodes. As shown in Figure 8a, during the initial discharge to 0.4 V, the content of Cu and Cu^1+^ increases significantly, and the content of Cu^2+^ decreases, which coincides with the XRD results that the peaks of Cu and Cu_2_O appear, and the peaks of CuO decrease. After charging to 1.05 V, Cu completely disappears, while the contents of Cu^2+^ and Cu^1+^ increase, which is also in line with the XRD results, where the peak of Cu_2_O increases and the peak of Cu disappears. Figure 8b shows that the content of Zn is high after the first and second discharges to 0.4 V, while it decreases after the first and second charges to 1.05 V, indicating that the charge carriers inside the battery are zinc ions. It is worth noting that the Coulombic efficiency of the batteries during the initial 30 cycles is lower than 100%, but it approaches 100% over the following 70 cycles (Figure 5). As shown in Figure S3, the XRD peak belonging to Cu_2_O is stronger and stronger as the number of cycles increases, and the valence of the Cu element is mainly +1 after 100 cycles. This indicates that the conversion reaction from CuO to Cu_2_O is ongoing during the initial 30 cycles. The main active material in the electrode is CuO at this stage. However, the reduction in CuO content makes the capacity decrease continuously. During the following cycles, more Cu_2_O is electrochemically activated, resulting in a gradual increase in capacity.

## 4. Conclusions

In conclusion, porous CuO microspheres were successfully synthesized using a simple hydrothermal reaction and were employed as a cathode material for AZIBs. The experimental results showed that the CuO-4h sample, after 4 h of hydrothermal reaction, exhibited the highest capacity and the longest lifecycle. At a current density of 500 mA g^−1^, a specific capacity of up to 131.7 mA h g^−1^ was maintained after 700 cycles with a capacity retention of 87.6%. In contrast, the electrochemical performance of the CuO-2h and CuO-6h samples was significantly inferior to that of the CuO-4h sample. This difference can be mainly attributed to the intact porous spherical structure and high specific surface area of CuO-4h. These characteristics facilitated effective contact between the active material and the electrolyte, thereby enhancing the charge transfer kinetics at the electrode/electrolyte interface. The ex situ XRD and ex situ XPS tests revealed that the CuO electrode underwent a conversion reaction during the initial charge/discharge cycle. In this process, CuO was reduced to Cu_2_O and Cu. The reaction was found to be partially reversible, with Cu being oxidized to Cu_2_O instead of CuO during the subsequent charging process. Throughout the following cycles, the active material participating in the reaction was primarily Cu_2_O. We believe that these findings can lay the groundwork for future modifications of copper-based materials and expand the options of cathode materials for AZIBs.

## Data Availability

The data that support the findings of this study are available from the corresponding authors upon reasonable request.

## Supplementary Materials

The following supporting information can be downloaded at: https://www.mdpi.com/article/10.3390/nano14131145/s1, Figure S1. (a) Cycling performance of the VGCF/Zn battery. (b) Cycling performance of the CuO-4h/Zn batteries using VGCF and Super P as the conductive additives; Figure S2. Nyquist plots of the CuO-2h, CuO-4h, and CuO-6h electrodes after 100 cycles; Figure S3. (a) Ex-situ XRD patterns and (b) ex-situ XPS spectra of the CuO-4h electrodes after different cycles (pristine, 1 cycle, 20 cycles and 100 cycles).
