# Supplementary material for: Porous CuO Microspheres as Long-Lifespan Cathode Materials for Aqueous Zinc-Ion Batteries
Source: Nanomaterials (Basel). 2024 Jul 3;14(13):1145. doi: 10.3390/nano14131145 (PMC11243631; doi:10.3390/nano14131145)
Supplement: Supplementary file 1 [file nanomaterials-14-01145-s001.zip › nanomaterials-3054797-supplementary.pdf]

Supporting Information

Porous CuO Microspheres as Long-Lifespan Cathode Materials for Aqueous Zinc-Ion Batteries

Yuqing Ai [1], Qiang Pang [1],* Xinyu Liu [1], Fangyun Xin [1], Hong Wang [1], Mingming Xing [1], Yao Fu [1] and Ying Tian [1]

[1] School of Science, Dalian Maritime University, Dalian 116026, P. R. China

* Correspondence: pangqiang@dlmu.edu.cn

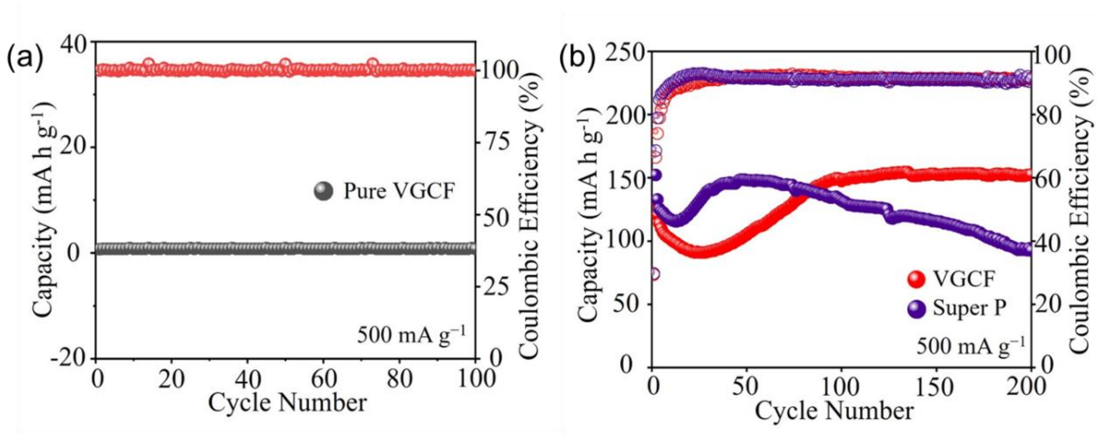

Figure S1. (a) Cycling performance of the VGCF/Zn battery. (b) Cycling performance of the CuO-4h/Zn batteries using VGCF and Super P as the conductive additives.

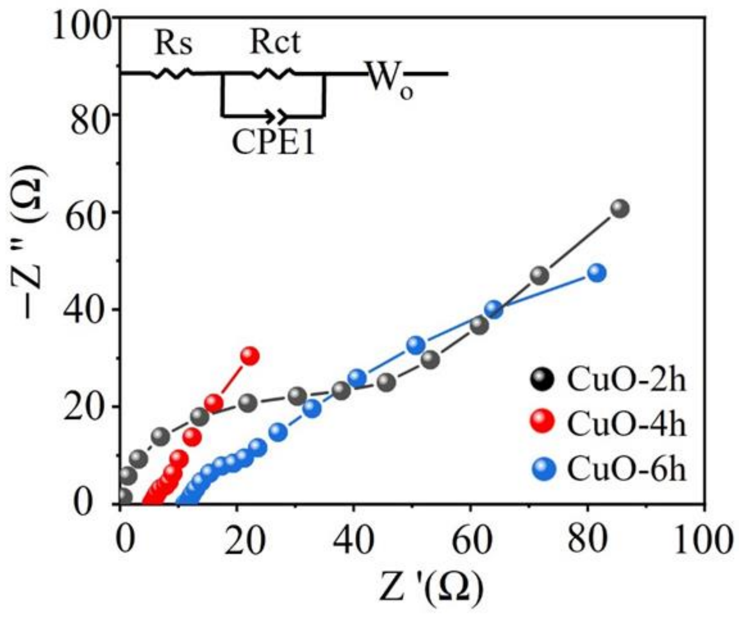

Figure S2. Nyquist plots of the CuO-2h, CuO-4h, and CuO-6h electrodes after 100 cycles.

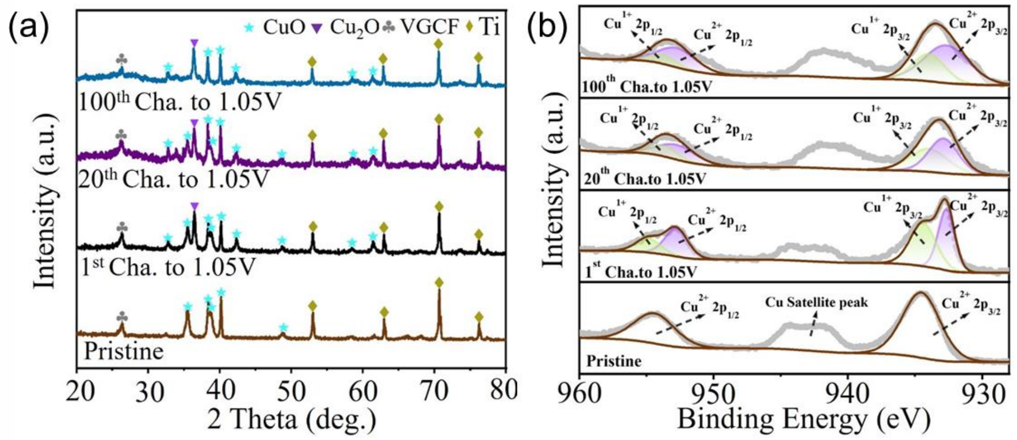

Figure S3. (a) Ex-situ XRD patterns and (b) ex-situ XPS spectra of the CuO-4h electrodes after different cycles (pristine, 1 cycle, 20 cycles and 100 cycles).
